# Enhancing Intrusion Detection for IoT and Sensor Networks Through Semantic Analysis and Self-Supervised Embeddings

**DOI:** 10.3390/s25227074

**Published:** 2025-11-20

**Authors:** Yanshen Liu, Yinfeng Guo

**Affiliations:** 1 Central South University, Changsha 410017, China; 2 Zhejiang Yu’an Information Technology Co., Ltd., Hangzhou 311121, China; guoyf@yuan-info.com

**Keywords:** intrusion detection system, feature engineering, data imbalance, sensor network security, IoT security

## Abstract

As cyber threats continue to grow in complexity and sophistication, the need for advanced network and sensor security solutions has never been more urgent. Traditional intrusion detection methods struggle to keep pace with the sheer volume of network traffic and the evolving nature of attacks. In this paper, we propose a novel machine learning-driven Intrusion Detection System (IDS) that improves intrusion detection through a comprehensive analysis of multidimensional data. Transcending traditional feature extraction methods, the system introduces geospatial context features and self-supervised semantic features that provide rich contextual information for enhanced threat identification. The system’s performance is validated on a carefully curated dataset from China Mobile, containing over 100 K records, achieving an impressive 98.5% accuracy rate in detecting intrusions. The results highlight the effectiveness of ensemble learning methods and underscore the system’s potential for real-world deployment, offering a significant advancement in the development of intelligent cybersecurity tools that can adapt to the ever-changing landscape of cyber threats. Furthermore, the proposed framework is extensible to IoT and wireless sensor networks (WSNs), where resource constraints and new attack surfaces demand lightweight yet semantically enriched IDS solutions.

## 1. Introduction

In the digital era, network security is no longer a luxury but a fundamental necessity. With the increasing sophistication of network threats, the task of securing modern communication infrastructures has become more challenging than ever. These threats, characterized by their complexity and intelligence, pose significant risks to the integrity and confidentiality of information systems. Detecting and neutralizing these malicious activities has thus become a pivotal concern in the realm of cybersecurity.Beyond traditional IT infrastructures, IoT and wireless sensor networks (WSNs) are now at the forefront of digital transformation. They support smart cities, industrial IoT, healthcare, and environmental monitoring. However, the characteristics of sensor networks—low-cost nodes, limited energy, and open wireless communication—make them highly vulnerable to a wide spectrum of attacks. Common threats include:Eavesdropping and Data Tampering, Sybil Attacks, Sinkhole and Blackhole Attacks, Energy-Depletion Attacks and False Data Injection.

The enormity of data generated in network environments renders manual inspection impractical. To bridge this gap, Intrusion Detection Systems (IDSs) have become indispensable [1]. These systems, designed to monitor and analyze data from diverse sources, play a critical role in identifying potential threats. When specific patterns or anomalies are detected, IDS triggers alerts for further investigation. This paper focuses on network-based IDS, which specializes in scrutinizing network traffic for signs of intrusion. The landscape of network-based IDS is varied, with distinctions mainly in the type of data analyzed and the methodologies employed for detection [2,3,4].

Historically, IDS relied on predefined signatures derived from expert knowledge to identify known threats [5]. However, with the advancement of technology, there has been a paradigm shift towards data-driven approaches, particularly those employing machine learning (ML) techniques [2]. These innovative methods empower IDS to identify not only known threats but also novel, sophisticated attacks that might elude traditional signature-based defenses. ML-based IDS are adept at learning from patterns in training data and applying this knowledge to detect previously unseen threats. Their autonomy in learning and decision-making significantly reduces the manual oversight required, thereby streamlining the management processes traditionally associated with IDS.

Despite these advancements, current intrusion detection systems grapples with complex and evolving network attacks, leading to high rates of false alarms [6,7,8]. This creates substantial strain on operational and maintenance efforts, rendering them less effective for network security situational awareness and security operation teams. Specifically, issues arise due to non-standardized alert formats across different software or devices, the overwhelming breadth and number of alerts laden with false positives, and varied alert classification standards that hinder quantifying system harm through attack alerts’ frequency and severity. Consequently, there is an imperative demand for innovative intrusion detection methodologies to augment the security and efficiency of threat judgment in networks.

Confronted with the intricate challenges of detecting network intrusions, this paper presents an innovative ML-based approach to enhance the identification process within network environments. Central to our methodology is the premise that accurate intrusion detection necessitates a comprehensive analysis of the multifaceted data contained within network logs, which often transcends the capabilities of traditional analysis techniques. Particularly challenging is the extraction and interpretation of features such as request bodies, which, while rich in semantic information, are typically represented as unstructured strings that are inherently complex for standard ML models to process. Despite the interpretative difficulties they present, these features hold critical contextual insights vital for the accurate detection of network threats. To address this, our research develops a comprehensive framework tailored for the analysis of multidimensional data. This framework is adept at discerning and processing a triad of feature categories: categorical, continuous, and semantic. Within this framework, semantic features, including request bodies and URL paths, are of particular interest. Our approach employs cutting-edge self-supervised learning techniques for embedding [9,10,11], which adeptly translate these complex string-based features into structured, numerical vectors. This advanced feature extraction methodology has demonstrated exceptional efficacy, seamlessly integrating with established ML classifiers to elevate the accuracy and adaptability of our IDS.

To validate our IDS, we collected a significant dataset from the network logs of China Mobile, one of China’s largest internet service providers (ISPs). This dataset, comprising over 100 K records, was carefully collected over a 14-day period through a meticulous manual labeling process. The authenticity of this dataset, derived from a real-world network environment, underlines its value and ensures its suitability for a comprehensive empirical evaluation of our IDS. The real-world context of the dataset not only bolsters its relevance but also enhances the applicability of our findings to practical network security scenarios. To assess the efficacy of our multidimensional feature extraction, we evaluated the features using various classifiers including K-Nearest Neighbors (KNNs), Decision Tree (DT), Support Vector Machine (SVM), Random Forest (RF), Naive Bayes (NB), and Linear Regression (LR). Our experimental results demonstrate the superiority of ensemble learning techniques in this context. The application of this method represents a significant enhancement in the realm of cybersecurity, enabling the development of more sophisticated and robust defense mechanisms against a wide array of network threats. Through this work, we contribute a robust analytical toolset to the cybersecurity community, offering a novel perspective on the utilization of complex data for the improvement of IDS. This paper summarizes our contributions as follows:

- We develop an innovative IDS system capable of detecting network threats with a 98.5% accuracy rate.
- We successfully utilize and interpret complex semantic features for enhanced intrusion detection.
- We comprehensively evaluate our IDS using a large-scale, real-world dataset from China Mobile, demonstrating the system’s effectiveness and practical applicability.

The structure of this paper is outlined as follows: Section 2 details the related works, providing context and background for our research. Section 3 delves into the foundational concepts of Natural Language Processing (NLP). Section 4 describes the design and architecture of our proposed system. In Section 5, we evaluate the performance of our system, and Section 6 concludes the paper, by summarizing our findings and contributions.

Furthermore, the geospatial context of network events serves as a crucial indicator of malicious intent. Attack patterns often exhibit geographical anomalies, such as login attempts from unexpected countries or coordinated attacks from specific regions. Traditional IDS often overlook this dimensionalty or treat IP addresses as opaque identifiers. Our approach explicitly models this geospatial context, extracting features like country and city from source IPs, and integrates them into a unified machine learning framework alongside semantic and other categorical features. This allows for the detection of threats that are not only semantically anomalous but also geospatially improbable, adding a powerful layer to our defense-in-depth strategy.

## 2. Related Work

The landscape of intrusion detection has witnessed a multitude of approaches aimed at augmenting the detection and classification of network anomalies. Traditional methods primarily relied on rules derived from expert knowledge and classical ML techniques. For instance, Snort, as explored by Bulajoul et al. [5], exemplifies an early approach that employs rule-based mechanisms for intrusion detection. In this system, data packets are scrutinized against a set of predefined rules, with non-conforming packets being discarded and detected intrusions triggering actions like logging and alerts, contingent on the nature of the detected activities. Another notable work, ATLANTIC [12], utilizes information theory principles to detect deviations in traffic flow entropy, combined with various ML algorithms for traffic classification. This method offers an innovative angle on identifying threats by analyzing entropy changes within flow tables.

Recent advancements have increasingly leaned towards deep learning-based solutions, diverging from conventional techniques reliant on expert-generated rules. These newer approaches aim to address complex dependencies in anomalies that traditional methods may overlook. Kim et al. [6] introduced a C-LSTM neural network that integrates CNN, LSTM, and DNN layers to analyze spatial-temporal dynamics in web traffic for anomaly detection. Complementing this, RawPower [7] developed a method for training a DNN directly with raw incoming byte streams, which eliminates the need for preprocessing and domain expertise, thus improving the model’s flexibility and adaptability. Addressing the limitations of traditional ML in real-time applications, Liu et al. [8] presented PL-CNN and PL-RNN approaches. These methods analyze payload data through convolutional and recurrent neural networks, respectively, learning feature representations directly from original payloads without manual feature extraction. Wang et al. [4] proposed the HAST-IDS, a system that automatically learns network traffic features using deep CNNs for spatial aspects and long short-term memory for temporal features. Naseer et al. [3] focused on anomaly detection models utilizing various deep neural network structures, including CNNs, autoencoders, and recurrent neural networks. Lastly, Liu et al. [2] proposed a novel feature engineering approach using raw packet payload data to construct block-based features, capturing both long-term and short-term dependencies in packet payload. This method also incorporates a neural network based on LSTM and CNN.

In addition to conventional IDS research, a growing body of work targets intrusion detection in IoT and sensor networks. A recent survey systematically reviews how *network-traffic fingerprinting* techniques are employed to identify and manage IIoT devices by analyzing flow-level statistics, protocol header fields, burstiness and periodicity patterns, and encrypted-traffic side signals [13]. These studies highlight the importance of lightweight anomaly detection mechanisms tailored to the computational and energy constraints of edge devices. However, most existing methods lack semantic feature modeling, which limits their ability to adapt to evolving and previously unseen attack scenarios.

In contrast to these existing methods, our work integrates self-supervised learning-generated deep embeddings with classic ML techniques. Our approach capitalizes on the benefits of deep learning while requiring only a minimal dataset for training. This efficiency is achieved by leveraging a well-trained embedding model pre-trained on a large dataset, enabling our system to extract meaningful features with minimal training data. Consequently, our IDS demonstrates both efficiency and effectiveness in detecting network intrusions. This combination of self-supervised deep embeddings with traditional ML classifiers offers a novel and efficient solution for intrusion detection. Consequently, the proposed IDS achieves both high efficiency and strong generalization capability across diverse network environments. To the best of our knowledge, this is the first IDS to systematically unify self-supervised semantic embeddings with geospatial and categorical context and ensemble classifiers into a single, resource-efficient framework deployable across enterprise and IoT settings.

## 3. Background: NLP and Advanced Embedding Techniques

In our IDS, we harness the capabilities of self-supervised learning-generated embeddings, an advanced technique in the field of NLP, which is a technology that empowers machines to comprehend, interpret, and engage with human languages, effectively bridging the gap between human communication and digital data processing.

### 3.1. Embedding in NLP

In the domain of ML, particularly for models processing textual data, numerical representation is crucial. Embeddings offer a solution for converting textual data into a format suitable for these models [14]. They are essentially vector representations of textual components, such as words, phrases, or entire sentences, within a continuous, multidimensional space. These embeddings are meticulously crafted to encapsulate rich semantic information, thereby capturing the subtle semantic nuances, contextual relevance, and syntactic relationships embedded in the text. Consequently, embeddings play an indispensable role in various ML tasks within NLP, offering a means to process and analyze text effectively.

### 3.2. Self-Supervised Learning in Embedding Generation

Recent advancements in embedding generation have seen a significant shift towards self-supervised learning methodologies, exemplified by models like Word2Vec [15]. This learning paradigm diverges from traditional supervised learning by generating its own training signals from the input data, negating the need for externally labeled datasets. This approach is particularly beneficial in the NLP field, where obtaining large-scale, annotated datasets can be challenging and resource-intensive. By capitalizing on the intrinsic structure of language data, self-supervised learning algorithms are able to cultivate comprehensive and nuanced text representations. Common training tasks for these algorithms include predicting missing words in sentences or identifying sentence sequences, facilitating a deeper understanding of language structure and semantics.

### 3.3. From Embedding to IDS

In this paper, we explore the application of advanced NLP embeddings to enhance network-based IDS. Our focus is on the effective interpretation of complex semantic features prevalent in network data. These features, rich in information, often present analysis challenges due to their inherently unstructured nature. Sentence embedding techniques [10,11], particularly utilizing state-of-the-art models like sentence transformers, are ideally suited for this purpose. We investigate how these advanced models can be effectively applied to network intrusion detection, enhancing our system’s ability to discern and interpret intricate semantic patterns within network data. This integration of sophisticated NLP techniques into IDS marks a significant stride in improving the system’s accuracy and efficiency in detecting network anomalies and threats.

Since our pipeline relies on sentence-level embeddings for URL paths and request bodies, we situate our design choices in light of recent large language model (LLM) surveys [16]. The proposed system adopts frozen, pre-trained embeddings to balance detection accuracy, computational efficiency, and privacy preservation. This approach eliminates the need for large-scale fine-tuning while maintaining strong few-shot generalization. Although frozen embeddings may exhibit reduced adaptability under domain shift (e.g., specialized network traffic), they enable lightweight and privacy-conscious deployment, as sensitive logs are not required for re-training. Overall, this integration of high-capacity semantic encoders with efficient learning modules provides a practical and scalable foundation for next-generation intrusion detection systems, aligning with best practices on cost, latency, and privacy trade-offs [16].

## 4. System Design

This section delineates the architecture of our IDS.

### 4.1. Design Overview

**Figure 1:**
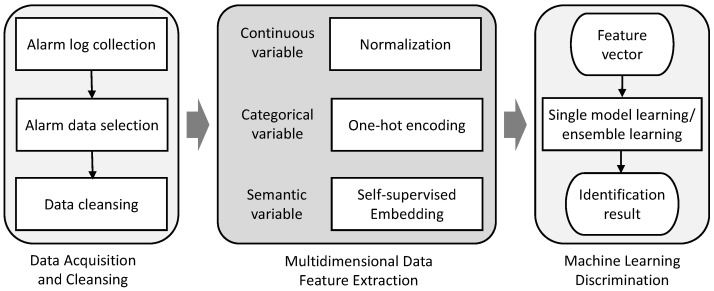
System overview.

The system’s architecture is divided into three core modules: Data Acquisition and Cleansing, Multidimensional Feature Extraction, and Machine Learning Discrimination, as shown in Figure 1. Each module plays a critical role in the process of identifying potential network threats.

Our IDS commences with the Data Acquisition and Cleansing module. This initial stage is dedicated to the systematic aggregation of alarm logs. The module’s primary responsibility is to extract pertinent features from these logs, followed by an extensive data cleansing procedure to ensure the integrity and applicability of the data. Table 1 exemplifies the output from the Data Acquisition and Cleansing module, illustrating the refined network event data ready for further processing.

Once the data is sanitized, it progresses to the Multidimensional Feature Extraction stage. Here, distinct network traffic attributes are meticulously converted into high-dimensional numerical vectors. This crucial step facilitates the transformation of unstructured raw data into an organized format, thereby capturing critical network behavior patterns and identifying potential anomalies. Within this module, features are categorized into continuous, categorical, and semantic types. Each category is treated with appropriate techniques: normalization for continuous variables, one-hot encoding for categorical variables, and self-supervised embedding for semantic variables.

The process culminates in the Machine Learning Discrimination module, which incorporates a comparative analysis using both single model and ensemble learning methodologies, such as SVM and RF classifiers. This module scrutinizes the feature vectors to pinpoint patterns indicative of network threats. Through a rigorous classification process, it distinguishes between normal and anomalous traffic, thereby enabling the prompt detection of intrusions. The discrimination module then evaluates the amalgamated multidimensional feature data, ascertaining the presence of anomalous traffic, and relays these findings to the network administrators for further action.

### 4.2. Feature Selection

Accurately characterizing alarm events is crucial, directly affecting whether classifiers are prone to overfitting or underfitting. To achieve better characterization, we designed a more general characterization method with strong universality.

The main features selected for alarm event vectorization include:

- Event start/end time: Records the start and end times of events for temporal analysis and comparison.
- Source and destination port numbers: Provide information related to network connections, aiding the analysis and discrimination of activities on specific ports.
- Total number of event occurrences: Counts the number of times each event occurs over a period to reveal the frequency and trend of the event.
- Source IP: Records the source IP address of the event for tracking and identifying potential attack sources.
- Source IP and Geospatial Context: Records the source IP address of the event. Crucially, we extend this to derive geospatial context features (e.g., country, city, AS number) for tracking and identifying potential attack sources based on geographical behavioral baselines.
- Request message: Divides the request message into various aspects, including content length, method, path, content type, user information, and main body. These details offer a comprehensive description of the request, aiding in the analysis and identification of potential threats.

Based on specific situations, we chose appropriate characterization methods to ensure the effectiveness and applicability of features. This method allows us to accurately describe and represent alarm events, providing a strong foundation for subsequent machine-learning discrimination methods.

### 4.3. Multidimensional Feature Extraction

In the Multidimensional Feature Extraction module, we undertake the intricate task of extracting a comprehensive feature set, pivotal for the effective performance of our IDS. The challenge here lies in the intricate nature of semantic variables, which contain rich contextual information and are inherently complex due to their unstructured format. Addressing this complexity, we first stratify the features into three fundamental categories: categorical variables, continuous variables, and semantic variables. This classification is crucial as it tailors the treatment and modeling of the diverse feature types according to their unique requirements.

- **Continuous Variables:** Continuous variables denote features with an infinite continuum of values, typically rendered as numerical quantities. In the context of alarm event characterization, this includes metrics such as the duration of events, total count of occurrences, source port numbers, request content length, etc. These variables are denoted by specific numerical values within the dataset.
- **Categorical Variables:** These variables represent features with a finite, discrete set of options, commonly expressed as labels or categories. Within the domain of alarm event characterization, examples of categorical variables are the request type (e.g., GET/POST), content type (e.g., text/html), user agent (e.g., Mozilla), and IP address, geolocation, which are confined to a predetermined spectrum of values in the dataset.
- **Semantic Variables:** Semantic variables refer to data entries rich in semantic content, like URL paths and request body strings. These variables are presented as specific strings within the dataset, which resist straightforward enumeration and pose substantial challenges for feature extraction and representation.

Owing to the inherent differences in their nature and types of values, categorical and continuous variables necessitate distinct processing approaches. Categorical variables are subjected to one-hot encoding to create a binary representation for each category within the model, while continuous variables are normalized to mitigate disparities in scale and to harmonize their influence during the modeling phase. Semantic variables, however, demand more sophisticated techniques to decipher and quantify the semantic meanings. By judiciously processing each type of feature, we maximize the extraction of pertinent information, thus fortifying the foundation for our subsequent machine-learning discrimination methods. This meticulous attention to the diverse data types is what sets our feature extraction process apart and significantly elevates the predictive prowess of our IDS.

#### 4.3.1. Continuous Variable Feature Extraction

For the continuous variables in our study, which include metrics such as duration of events, total count of occurrences, source port numbers, and request content length, we employ robust normalization techniques. Normalization is a critical step in preparing continuous variables for modeling, and it’s achieved through the following transformation:

(1) $$v^{\prime }=\frac{v-\min(V)}{\max(V)-\min(V)}$$

where *v* is the original value, *V* is the set of all values for the variable, and $v^{\prime }$ is the normalized value. This formula scales the range of continuous variable values to a standard interval, typically [0, 1]. This method mitigates the discrepancies in scale among different variables, ensuring they exert comparable influence in the predictive modeling process, thereby maintaining uniformity and importance across all continuous variables.

#### 4.3.2. Categorical Variable Feature Extraction

To manage the categorical variables within our dataset, which encompass elements such as request type (e.g., GET, POST), content type (e.g., text/html), user agent (e.g., Mozilla), and IP address, we employ the technique of one-hot encoding. The utilization of one-hot encoding for categorical variables is imperative for several reasons. Primarily, one-hot encoding converts each category value into an exclusive binary vector. This process involves generating a distinct binary feature for every possible category, assigning a value of ‘1’ to indicate the presence of a category, and setting all other elements to ‘0’. This binary transformation is mathematically denoted by the following representation:

(2) $$x_{i}=\left\{\begin{array}{cc} 1 & \mathrm{if}\;v=c_{i}\\ 0 & \mathrm{otherwise} \end{array}\right.$$

where $x_{i}$ is the binary feature for category $c_{i}$ and *v* is the original value of the variable. This encoding method is particularly potent because it ensures the non-ordering of categorical variables, providing solely presence or absence information. This characteristic is crucial as it prevents the introduction of artificial ordinal relationships among categories, which could potentially mislead the modeling process.

We implement one-hot encoding on various categorical data elements. Notably, for source IPs, we initially utilize a static IP map to retrieve geographic locations. In cases where this approach is unsuccessful, we resort to dynamic queries to acquire country and city features, which are then subjected to one-hot encoding. Similarly, for request methods, content types, and user information, we enumerate each occurring type and proceed with one-hot encoding. For instance, common request methods like GET, POST, PUT, and DELETE, among others, total 16 types, thereby necessitating a 16-bit one-hot encoding scheme. This meticulous approach to variable embedding ensures that our model comprehensively understands and accurately interprets the complex nature of the network data, thereby enhancing the predictive accuracy and robustness of our IDS.

#### 4.3.3. Semantic Variable Feature Extraction

In our pursuit to extract meaningful features from semantic variables, such as URL paths and request body strings, we incorporate the self-supervised learning methods for embedding [10]. Our approach utilizes Transformer [17] that rely on the self-attention mechanism to transform these semantic variables into high-dimensional vectors with real-value encoding.

**Embedding Model Backbone.** The Transformer, as conceptualized by Vaswani et al. [17] in the foundational paper “Attention is All You Need,” encodes contextual information for input tokens. Input vectors $x_{i}{i=1}^{\left|x\right|}$ are assembled into an initial matrix $H^{0}=\left[x_{1},\ldots ,x\left|x\right|\right]$. The transformation process through successive Transformer layers is defined by the relation:

(3) $$H_{l}^{\prime }={\mathrm{Transformer}}_{l}\left(H^{l-1}\right),\;l\in \left[1,L\right]$$

Here, *L* denotes the total number of Transformer layers, with $H^{L}=\left[h_{1}^{L},\ldots ,h_{\left|x\right|}^{L}\right]$ representing the output of the final layer. Each element $h_{i}^{L}$ serves as a contextualized representation of the corresponding input $x_{i}$. A Transformer layer is composed of a self-attention sub-layer paired with a fully connected feed-forward network. This architecture is fortified by residual connections, as introduced by He et al. [18], and is followed by layer normalization techniques proposed by Ba et al. [19].

Each Transformer layer employs multiple self-attention heads to synthesize the outputs from the preceding layer. For the *l*-th layer, the output from self-attention head $AO_{l,a}$, for $a\in [1,A_{h}]$, is computed using the following expressions:

(4) $$Q_{l,a}=H^{l-1}W_{l,a}^{Q},\;K_{l,a}=H^{l-1}W_{l,a}^{K},\;V_{l,a}=H^{l-1}W_{l,a}^{V}$$

(5) $$A_{l,a}=\mathrm{softmax}\left(\frac{Q_{l,a}K_{l,a}^{T}}{\sqrt{d_{k}}}\right)$$

(6) $$AO_{l,a}=A_{l,a}V_{l,a}$$

The output of layer $H^{l-1}$ is linearly projected to queries (*Q*), keys (*K*), and values (*V*) through the weight matrices $W_{l,a}^{Q},W_{l,a}^{K},W_{l,a}^{V}$. The attention distribution $A_{l,a}$ is derived from the scaled dot-product of queries and keys. The model employs $A_{h}$ self-attention heads, with the product $d_{k}\times A_{h}$ equating to the hidden dimension $d_{h}$ found in BERT models [14].

Based on the uncased MiniLM architecture, our transformer model features 12 layers with a hidden size of 384, 12 attention heads, and encompasses 33 million parameters [20]. It is initialized with the pre-trained weights from the MiniLM implementation [21].

**Learning Embedding through Self-Supervised Learning.** Employing self-supervised contrastive learning objectives within siamese and triplet network structures [22], the embedding model is fine-tuned on a dataset of 1 billion sentence pairs. The contrastive learning task challenges the model to discern the correct pairing from a randomly selected sentence set. The product of this learning process is a suite of dense, 384-dimensional vectors that embody the rich semantic details of the sentences [10]. The fine-tuned model is available on Hugging Face [23]. **Embedding Dimension Compression.** Upon receiving the embeddings from the refined model, we confront the issue that semantic features often reside within a *lower-dimensional manifold* of the expansive 384-dimensional space. To address this, we utilize Principal Component Analysis (PCA) for dimensionality reduction [24]. PCA efficiently isolates and retains the most salient information, compacting the high-dimensional embeddings into a more manageable form for subsequent ML applications. This compression is instrumental in exploiting the wealth of information within semantic variables and furnishing potent, expressive features for the discriminative mechanisms of our ML algorithms.

### 4.4. Machine Learning Discrimination Algorithms

The pivotal component of our network security detection framework involves associating extracted features with the nature of network traffic, differentiating between benign and malicious activities. Conventional methods, anchored in expert knowledge and static rules, have faltered against the dynamic nature of cyber threats. The integration of ML into this domain offers a promising alternative, enabling more adaptive and effective traffic discrimination.

#### 4.4.1. ML Classifiers

We utilize several ML classifiers, each with its unique approach to pattern recognition within network traffic.

**Support Vector Machine:** SVMs operate on the principle of finding a hyperplane in an N-dimensional space that distinctly classifies the data points. The optimal hyperplane is determined by the equation:

(7) $$\max_{w,b}\frac{1}{\left||w|\right|}\;\mathrm{subject}\;\mathrm{to}\;y_{i}\left(w\cdot x_{i}+b\right)\geq 1,\forall i$$

Here, *w* represents the weight vector perpendicular to the hyperplane, *b* is the bias that adjusts the hyperplane’s position, $x_{i}$ denotes the feature vectors, $y_{i}$ are the corresponding class labels, and ||w|| is the norm of the weight vector, indicating the inverse of the margin. In our implementation, we employ SVM with RBF kernel, regularization parameter C=1.0, and kernel coefficient γ = ‘scale’.

**Linear Regression Classifier:** This classifier applies LR to classification problems. It predicts the target by fitting the best linear relationship between the feature vector and the target label. The implementation uses default scikit-learn parameters with fit_intercept=True and solver=‘lbfgs’.

**Naive Bayes Classifier:** Based on Bayes’ Theorem, this classifier assumes the independence of features and calculates the probability of a label given a set of features using the formula:

(8) $$P\left(Y|X\right)=\frac{P(X|Y)P(Y)}{P(X)}$$

Here, P(Y|X) is the probability of label *Y* given feature set *X*, P(X|Y) is the likelihood of feature set *X* given label *Y*, P(Y) is the prior probability of label *Y*, and P(X) is the prior probability of feature set *X*. We use Gaussian Naive Bayes with default variance smoothing parameter var_smoothing = $10^{-9}$.

**K-Nearest Neighbors:** KNN works on the principle of feature similarity, classifying a data point based on how closely it resembles the other points in the training set. Our KNN implementation uses k=5 neighbors with Euclidean distance metric (metric=‘minkowski’, p=2) and uniform weighting (weights=‘uniform’).

**Decision Tree:** DTs employ a tree-like model of decisions. The goal is to learn decision rules inferred from data features, represented by the tree branches, leading to conclusions about the target value. We configure the DT with Gini impurity criterion (criterion=‘gini’), no maximum depth constraint (max_depth=None), minimum samples split of 2 (min_samples_split=2), and minimum samples per leaf of 1 (min_samples_leaf=1).

**Ensembled Classifier: Random Forest.** To overcome the limitations of single-model ML algorithms, we explore the efficacy of ensemble learning algorithms. Such algorithms, particularly RF, construct a collective model from multiple DTs to mitigate bias and variance:

(9) $$\mathrm{Random}\;\mathrm{Forest}=\frac{1}{N}\sum_{i=1}^{N}DT_{i}\left(X\right)$$

where *N* is the number of trees and $DT_{i}$ is the *i*th DT’s prediction. The dual sources of randomness in RFs—both in the selection of the bootstrap samples and the feature subsets for splitting—significantly reduce the variance of the model. This is achieved without substantially increasing the bias, leading to a model that is both accurate and robust against overfitting. Our RF implementation uses n_estimators=100 trees, square root of feature count for maximum features (max_features=‘sqrt’), no maximum depth constraint (max_depth=None, balanced class weights (class_weight=‘balanced’), and bootstrap sampling enabled (bootstrap=True). In contrast to standard approaches, our implementation harnesses the probabilistic predictions of individual classifiers, combining them through averaging rather than majority voting. This nuanced approach not only bolsters the accuracy of traffic discrimination but also provides a more comprehensive understanding of the underlying data patterns. We also compare with modern ensembles, including XGBoost/LightGBM/CatBoost in Section 5.2.

#### 4.4.2. Data Balancing

The imbalance in the dataset, characterized by a significantly higher proportion of benign instances compared to attack instances, can bias the ML classifier towards favoring the benign class [25]. This skew in the class distribution often results in a model that is more adept at predicting the majority class while misclassifying the minority class, which, in the context of intrusion detection, is often the more critical one to identify correctly. To rectify this imbalance and enhance the classifier’s ability to detect attacks, we employ a downsampling technique. Downsampling involves reducing the number of instances from the majority class to match the minority class, thereby equalizing the class distribution. This technique ensures that during the training phase, the ML classifier is not overwhelmed by the majority class and can learn to recognize the patterns of both benign and attack data more effectively. As a result, downsampling is a crucial step in preparing our dataset for the training of a more balanced and effective intrusion detection model.

## 5. Evaluation

We now embark on a detailed evaluation of our IDS.

### 5.1. Experimental Setup

**Dataset Collection.** To rigorously assess our IDS, we leveraged a large-scale dataset collected by China Mobile, one of the largest ISPs in China. This dataset is not only substantial, with more than 100 K records, but also profoundly valuable due to its derivation from an authentic network environment. Over a 14-day period, the data was subjected to a thorough manual labeling process, underscoring the dataset’s practical significance and ensuring its exceptional quality for the empirical evaluation of the IDS. The real-world origins of the dataset augment its applicability and enhance the relevance of our findings to actual network security scenarios.

**Implementation.** The system was implemented using Python version 3.9.5 and SentenceTransformers framework [26], with PyTorch2.1 serving as the foundational framework for developing the ML models. The computational experiments were carried out on a machine running the Ubuntu 22 operating system, chosen for its stability and support for the necessary computational libraries.

Semantic features were extracted using the all-MiniLM-L6-v2 sentence embedding model, which outputs 384-dimensional vectors for both request path and request body text. To reduce computational complexity while preserving discriminative information, we applied Principal Component Analysis (PCA) with Maximum Likelihood Estimation (MLE) to automatically determine the optimal number of components. PCA was fit exclusively on the training split and subsequently applied to the validation and test sets to avoid information leakage. Since SentenceTransformer embeddings are already normalized and centered, no additional standardization was performed prior to PCA. For request path embeddings (384 dimensions), PCA with MLE selected 190 components, explaining 95% of the variance; for request body embeddings, 195 components were retained, explaining 94% of the variance.

**Metrics.** The efficacy of our classifiers in discriminating network traffic was assessed using two key metrics: Accuracy and the F1 Score. Accuracy is defined as:

(10) $$\mathrm{Accuracy}=\frac{TP+TN}{TP+FP+FN+TN}$$

where TP is the number of true positives, TN is the number of true negatives, FP is the number of false positives, and FN is the number of false negatives. This metric measures the overall correctness of the classifier and ranges from 0 (worst) to 1 (best).

The F1 Score is defined as:

(11) $$F1\;\mathrm{Score}=2\times \frac{\mathrm{Precision}\times \mathrm{Recall}}{\mathrm{Precision}+\mathrm{Recall}}$$

where Precision and Recall are defined as:

(12) $$\mathrm{Precision}=\frac{TP}{TP+FP}$$

(13) $$\mathrm{Recall}=\frac{TP}{TP+FN}$$

The F1 Score is the harmonic mean of Precision and Recall, providing a single metric that balances both the false positives and false negatives.

Additionally, we report ROC-AUC (Area Under the Receiver Operating Characteristic Curve) and PR-AUC (Area Under the Precision-Recall Curve). ROC-AUC measures the classifier’s ability to distinguish between classes across all classification thresholds, while PR-AUC is particularly informative for imbalanced datasets as it focuses on the positive class performance. We also report Recall@95% Specificity and Recall@99% Specificity, which indicate the detection rate when the false positive rate is constrained to 5% and 1%, respectively. These metrics are critical for real-world deployment where minimizing false alarms is essential.

### 5.2. Overall Performance

We evaluate the overall performance of our IDS. Features are extracted using our proposed method, and we compare the performance of different classifier algorithms in discriminating network traffic. This includes classical methods (SVM, KNN, LR classifier, NB classifier, Decision Tree, and RF classifier) as well as modern gradient boosting baselines (XGBoost, LightGBM, and CatBoost). Table 2 presents a comprehensive comparison of all evaluated classifiers across multiple metrics.

As shown in Table 2, classical methods show varied performance. SVM has the lowest accuracy and F1 Scores, both below 66%. The performance of NB is slightly better than SVM but still below 67%. Both KNN and LR exceed 70% in accuracy and F1 Scores but are below 80%, making them challenging to use in practical scenarios. Decision Tree performs better at 96.26% accuracy.

Among these classical approaches, Random Forest (RF) stands out, attaining 99.32% accuracy and F1-score. Notably, modern gradient boosting methods such as LightGBM and CatBoost achieve marginally higher accuracy (up to 99.66%) and perfect precision or recall. All three top-performing ensemble methods (RF, LightGBM, CatBoost) reach perfect ROC-AUC (99.9%) and maintain over 99.9% recall at 99% specificity, reflecting exceptional discrimination capability with minimal false alarms.

Three key takeaways emerge from our evaluation:

1. **Ensemble Methods Excel with Our Features.** Both classical ensemble (RF) and modern gradient boosting methods (XGBoost, LightGBM, CatBoost) demonstrate superior performance compared to single classifiers, aligning with the effectiveness of our feature extraction design. This emphasizes the compatibility between our feature extraction method and ensemble-based classifiers.
2. **Modern Baselines Confirm Feature Quality.** The fact that multiple state-of-the-art methods (RF, LightGBM, CatBoost) all achieve near-perfect performance (>99%) validates the discriminative power of our extracted features, rather than being an artifact of a specific classifier.
3. **Practical Deployment Viability.** While gradient boosting models achieve slightly higher accuracy, RF provides a more favorable trade-off between performance and operational efficiency. It requires significantly less hyperparameter tuning, offers faster inference, and incurs lower computational and memory overhead during both training and deployment. These properties make RF particularly suitable for real-time or resource-constrained environments (e.g., edge gateways or IoT intrusion detection), where lightweight yet highly accurate models are essential.

### 5.3. Effect of Size of Training Set

**Figure 2:**
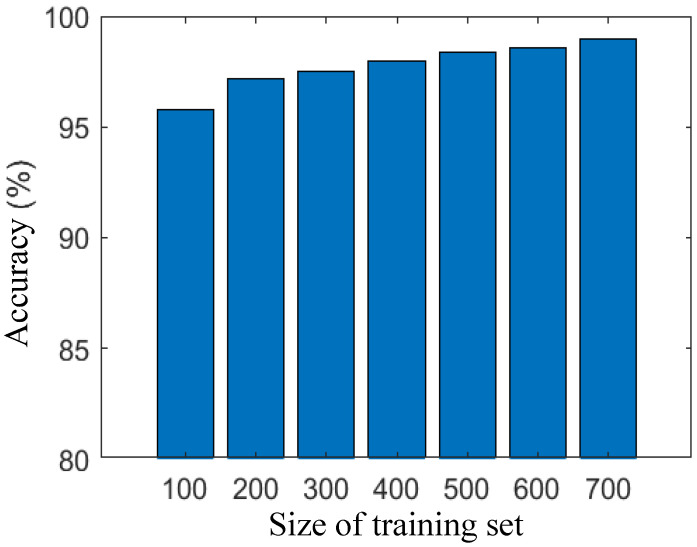
Accuracy under different sizes of training sets.

**Figure 3:**
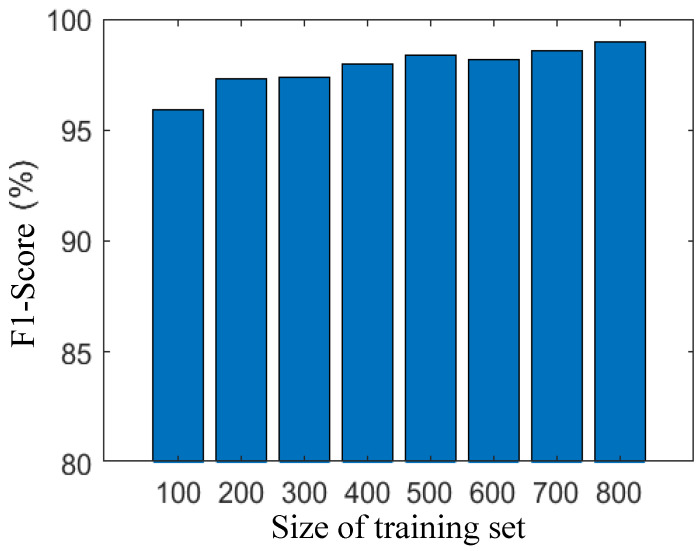
F1-score under different sizes of training sets.

Our semantic analysis-based IDS excels in feature extraction from the training set, demonstrating efficacy even with a relatively small training set. In this study, we investigate the influence of training set size, specifically the number of training samples, on the performance of network traffic discrimination. To assess the impact, we systematically increased the number of training sets in increments of 100, ranging from 100 to 800 samples. Subsequently, we measured the accuracy and F1 Scores at each step. As depicted in Figure 2 and Figure 3, both accuracy and F1 Scores exhibit a positive correlation with the size of the training set. Remarkably, even with a modest training set of 100 data points, our IDS achieves high accuracy and F1 Scores, reaching 95.8% and 95.9%, respectively. As the training set expands to 400 samples, both accuracy and F1 Scores surpass 98%. With a larger training set size of 800, the accuracy and F1 Scores further improve, reaching 98.5%.

These findings underscore the robustness and adaptability of our semantic analysis-based IDS, showcasing its ability to deliver exceptional discrimination performance across a range of training set sizes. The results affirm the system’s capability to effectively leverage semantic analysis for feature extraction, making it a resilient and efficient solution for network intrusion detection.

### 5.4. Effect of Number of Trees

**Figure 4:**
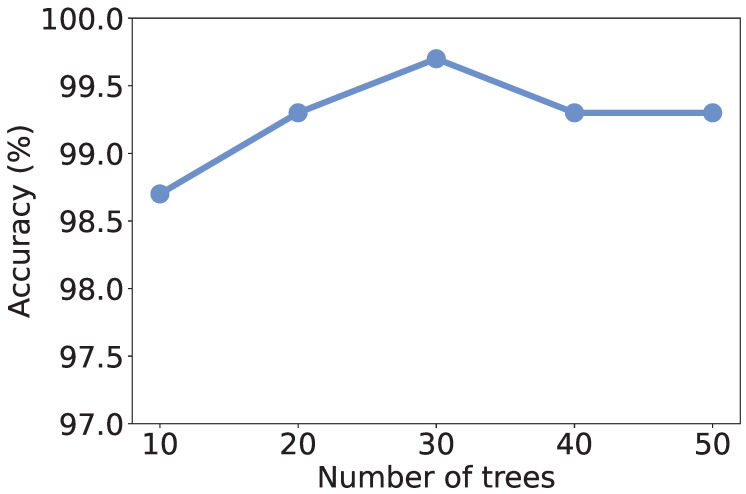
Accuracy under different numbers of trees.

**Figure 5:**
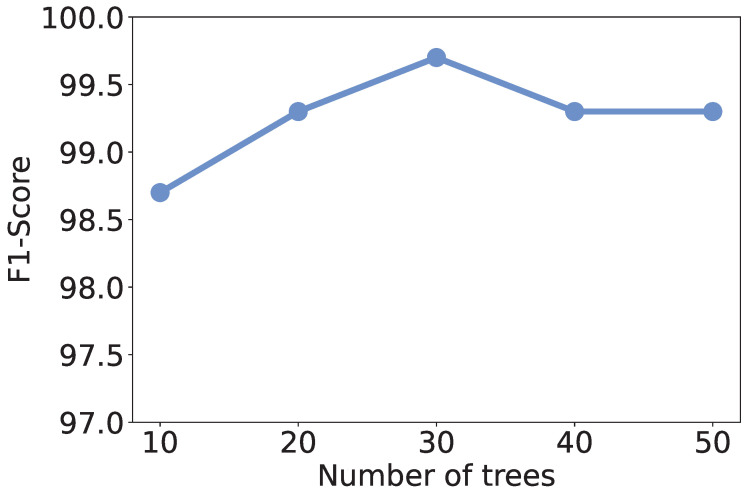
F1-score under different numbers of trees.

**Figure 6:**
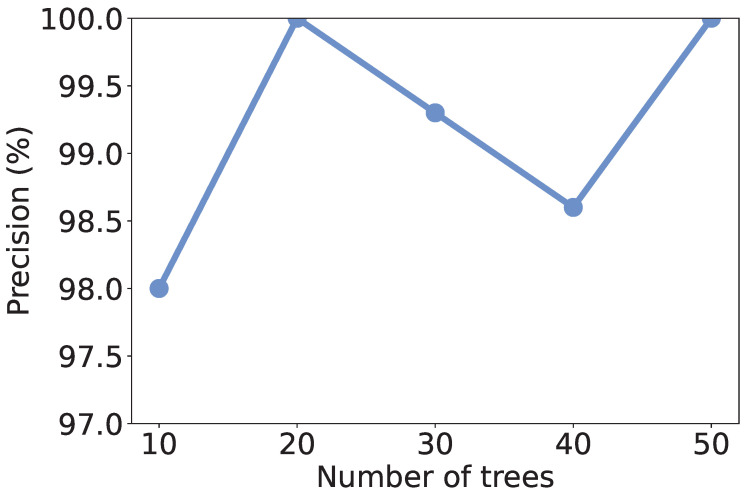
Precision under different numbers of trees.

**Figure 7:**
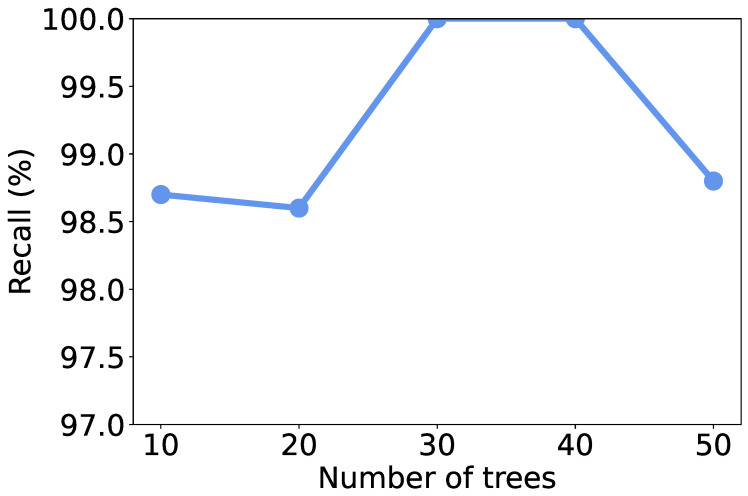
Recall under different numbers of trees.

Recall that we use RF as the learning model, the number of trees in the model may affect the discrimination performance. To investigate the effect of the number of trees, we vary it from 10 to 50 and recalculate the metrics. As shown in Figure 4, Figure 5, Figure 6 and Figure 7, all the metrics are within [98%, 100%]. When only 10 trees are employed, accuracy, F1-score, and precision reach their lowest values, respectively. But they are still higher than 98%, meaning that a small number of trees is sufficient to achieve decent traffic identification performance. Meanwhile, the variation trends of accuracy, F1-score, and recall are similar to each other, i.e., first increase and then decrease. When there are 30 trees, these three metrics show the highest values. Thus, we suggest the users use 30 trees to build an RF classifier.

### 5.5. Exploring the Significance of Features in IDS

In this experiment, we delve into the significance of each type of feature to comprehensively understand their impact on the performance of our IDS. We systematically evaluate the system’s performance under the influence of a single type of feature, shedding light on their individual contributions. To ensure statistical rigor, we keep all preprocessing constants fixed and perform controlled ablations isolating each feature type. Specifically, we separate the geolocation attribute from other categorical features to assess its individual impact. Each experiment is repeated across ten random data splits to compute the mean and 95% confidence intervals (CIs).

**Figure 8:**
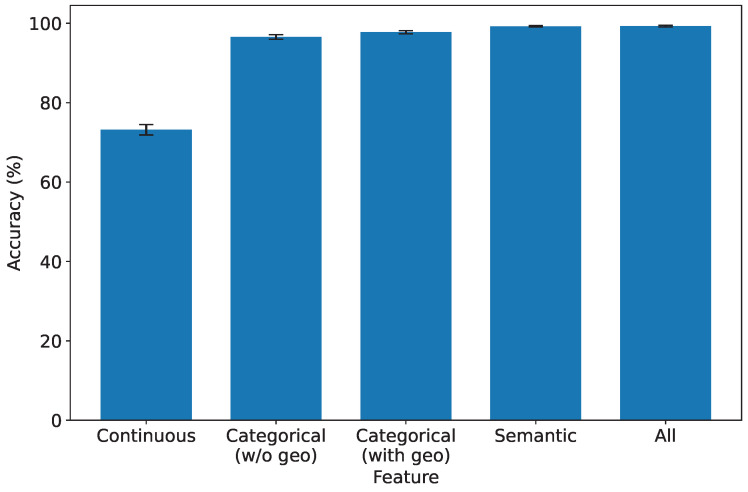
Accuracy under different types of features.

**Figure 9:**
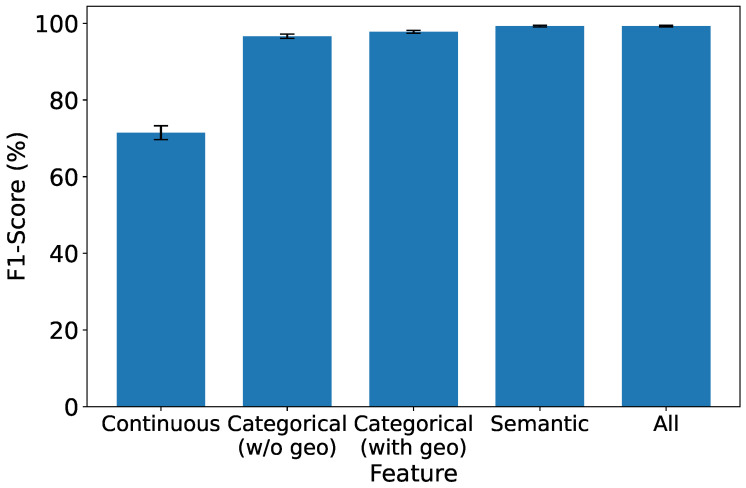
F1-score under different types of features.

**Figure 10:**
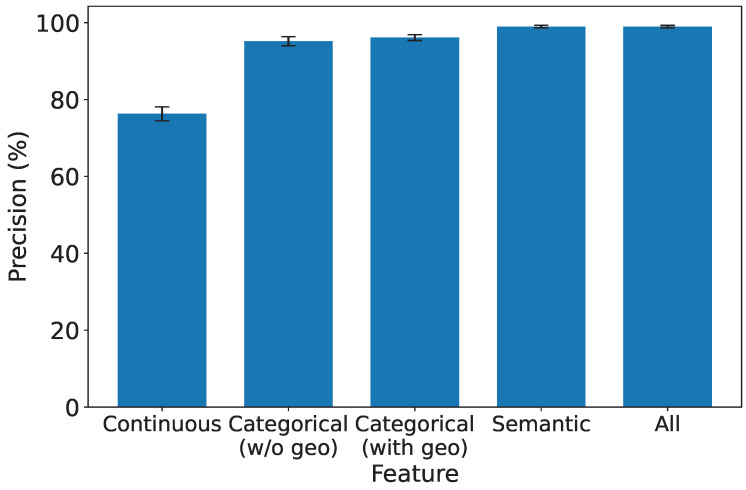
Precision under different types of features.

**Figure 11:**
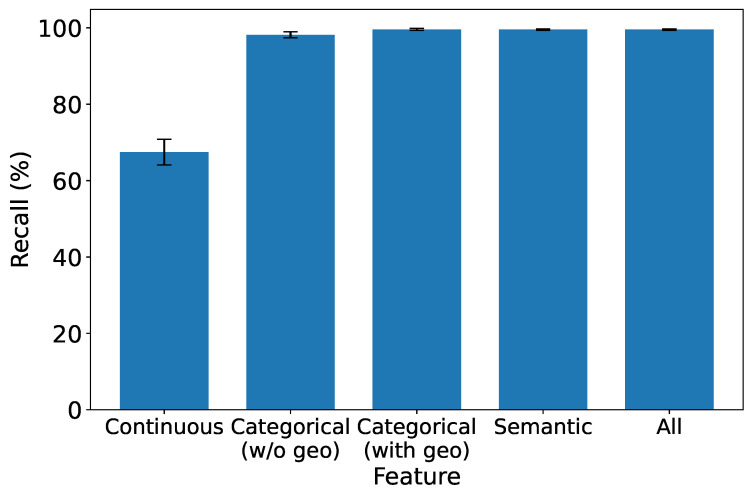
Recall under different types of features.

As depicted in Figure 8, Figure 9, Figure 10 and Figure 11, which report accuracy, F1, precision, and recall along with their 95% confidence intervals, continuous features exhibit the lowest performance across all metrics. Conversely, both categorical and semantic features showcase commendable performance, consistently surpassing 90%. Notably, incorporating geolocation within categorical features further improves detection performance. Three key insights emerge from our analysis:

1. **Effectiveness of Semantic and Categorical Features.**
  - *Semantic Features:* Achieve an average accuracy of 98% with narrow CIs across all metrics, highlighting their effectiveness in capturing meaningful contextual information from request paths and bodies.
  - *Categorical Features:* Attain 96.6% (±0.6%) accuracy without geolocation and improve to 97.8% (±0.4%) when geolocation is included. This demonstrates that spatial attributes enhance traffic characterization in multi-region deployments.
2. **Effective Feature Fusion.** When all types of features are employed collectively, our IDS achieves optimal performance (99.3% ±0.2% accuracy). The seamless combination of our feature extraction methods contributes synergistically to the final result, underscoring the benefit of heterogeneous information integration.
3. **Extra Robustness Through Feature Diversity.** In scenarios where categorical features may lack robustness, the inclusion of stable semantic embeddings provides complementary guidance. This diversity enhances system resilience, ensuring effective intrusion detection even under varying traffic distributions or unseen attack patterns.

These insights highlight the importance of feature diversity and the well-designed fusion of continuous, semantic, and categorical features in enhancing the overall performance and robustness of our IDS. For instance, in our dataset, over 15% of the detected brute-force attacks originated from geolocations that exhibited no prior legitimate user activity, a pattern efficiently captured by our model’s geospatial features. This demonstrates a clear advantage over systems that lack such contextual awareness.

## 6. Conclusions

This paper presents a machine learning-based Intrusion Detection System designed to effectively manage the complexities of multidimensional network data. By incorporating self-supervised learning for semantic variable embedding, the system transforms unstructured textual information into structured numerical representations. This approach enables the model to jointly process continuous and categorical variables, exhibiting particular strength in interpreting complex semantic and geospatial information that conventional systems often fail to capture. The fusion of semantic and geospatial anomaly detection proves to be a powerful combination for identifying evolving network threats.

Beyond conventional enterprise networks, the proposed system demonstrates strong applicability in IoT and sensor environments, where semantic enrichment and lightweight deployment are crucial for maintaining robust security under resource constraints. Comprehensive validation using a large-scale real-world dataset from China Mobile confirms that the system accurately detects diverse network threats while maintaining practical deployability.

While the current implementation achieves high accuracy and robustness, future extensions may focus on adaptive domain generalization and incremental model updates to cope with continuously evolving traffic patterns and unseen attack types. In addition, integrating advanced embedding models or federated learning mechanisms could further enhance scalability and privacy preservation across distributed infrastructures.

By tightly integrating innovative representation learning with efficient ensemble-based classification, the proposed IDS effectively addresses the dual challenges of multidimensional data processing and semantic understanding, contributing a practical and extensible foundation for next-generation intrusion detection in both enterprise and IoT ecosystems.

## Figures and Tables

**Table 1:** An example of processed network event data from the Data Acquisition and Cleansing module.

Field	Value
Generation Time	23 March 2023 11:26
End Time	23 March 2023 11:26
Source IP	124.220.174.243
Source Port	58306
Destination IP	120.199.235.18, Zhejiang, China
Destination Port	37020
Device Source	192.168.2.1
Occurrence Count	1
Request Message	GET/HTTP/1.1 Host: zzjdhgl.zj.chinamobile.com:37020 User-Agent: Mozilla/5.0 (X11; Linux x86_64) AppleWebKit/537.36 (KHTML, like Gecko) Chrome/34.0.1847.137 Safari/4E423F Connection: close Accept: image/gif, image/x-xbitmap, image/jpeg, image/pjpeg, image/png, */* Accept-Charset: iso-8859-1,utf-8;q=0.9,*;q=0.1 Content-Type: %[#context[’ISOP#@!STARTcom.opensymphony.xwork2ISOP#@!END. dispatcher.HttpServletResponse’].addHeader(’X-Hacker’,’Bounty Plz’)].multipart/form-data Accept-Encoding: gzip

**Table 2:** Comprehensive performance comparison of classical and modern classifiers. All metrics reported as percentages (%).

Classifier	Accuracy	Precision	Recall	F1	ROC-AUC	PR-AUC	R@95%S	R@99%S
*Classical Classifiers*								
KNN	77.21	80.23	72.30	76.06	86.02	84.57	55.48	23.29
SVM	65.65	68.18	58.90	63.24	71.29	68.95	34.25	9.59
Logistic Reg.	73.21	75.34	69.18	72.15	80.45	78.92	48.63	17.81
Naive Bayes	66.78	69.86	60.27	64.76	72.58	70.34	35.62	10.96
Decision Tree	96.26	96.58	95.89	96.23	96.26	96.18	92.47	84.93
*Ensemble Classifier*								
Random Forest	99.32	99.32	99.32	99.32	99.99	99.99	99.99	99.99
*Modern Gradient Boosting Baselines*								
XGBoost	98.30	99.32	97.26	98.28	99.93	99.92	98.63	94.52
LightGBM	99.66	99.99	99.32	99.66	99.99	99.99	99.99	99.99
CatBoost	99.66	99.32	99.99	99.66	99.99	99.99	99.99	99.99

R@95%S = Recall at 95% Specificity; R@99%S = Recall at 99% Specificity.

## Data Availability

The original contributions presented in this study are included in the article. Further inquiries can be directed to the corresponding author.

## Abbreviations

The following abbreviations are used in this manuscript:

IDS	Intrusion detection system
ML	Machine learning
ISP	Internet service provider
URL	Uniform resource locator
NLP	Natural language processing
IP	Internet protocol
CNN	Convolutional neural network
DNN	Deep Neural Network
LSTM	Long short-term memory
SVM	Support vector machine
RF	Random forest
NB	Naive Bayes
LR	Linear regression
KNN	K-nearest neighbors
DT	Decision Tree
